# Voltammetric Studies on Gold Electrodes Coated with Chitosan-Containing Layer-by-Layer Films

**DOI:** 10.3390/ma6115427

**Published:** 2013-11-21

**Authors:** Shigehiro Takahashi, Ryota Watahiki, Kohji Tomida, Baozhen Wang, Jun-ichi Anzai

**Affiliations:** 1 Graduate School of Pharmaceutical Sciences, Tohoku University, Aramaki, Aoba-ku, Sendai 980-8578, Japan; E-Mails: t-shigehiro@m.tohoku.ac.jp (S.T.); b2ym1036@s.tohoku.ac.jp (R.W.); a8yb1058@s.tohoku.ac.jp (K.T.); 2 Department of Nutrition and Food Hygiene, School of Public Health, Shandong University, 44 WenhuaXilu, Jinan, Shandong 250012, China; E-Mail: bzhenw@hotmail.com

**Keywords:** polysaccharide, chitosan, layer-by-layer film, cyclic voltammetry, hexaammine ruthenium ion, ferricyanide ion

## Abstract

Gold (Au) electrodes coated with layer-by-layer (LbL) thin films composed of chitosan (CHI) were prepared to evaluate the redox properties of hexaammine ruthenium ions, Ru(NH_3_)_6_^3+^, and ferricyanide ions, Fe(CN)_6_^3−^ LbL films were prepared on an Au electrode by electrostatic LbL deposition using polycationic CHI and poly(vinyl sulfate) (PVS) or poly(acrylic acid) (PAA) as anionic component. Redox peak current in cyclic voltammetry of Ru(NH_3_)_6_^3+^ on the CHI/PVS and CHI/PAA film-coated electrodes increased with increasing thickness of the films. Interestingly, the cyclic voltammograms showed two pair of redox peaks, originating from Ru(NH_3_)_6_^3+^ diffusing across the LbL layers and from those confined in the film. The results were rationalized in terms of the electrostatic interactions between Ru(NH_3_)_6_^3+^ and excess negative charges in the LbL films originating from PVS and PAA. In contrast, Fe(CN)_6_^3−^ was not confined in the LbL films due to electrostatic repulsion of Fe(CN)_6_^3−^ and excess negative charges. Significant amounts of Ru(NH_3_)_6_^3+^ were confined in the films at pH 7.0, whereas few ions were bound at pH 3.0 due to the reduced net negative charge in the films. The results suggest a potential use of the CHI-containing LbL films as scaffold for immobilizing positively charged ionic species on the electrode surface.

## 1. Introduction

Development of modified electrodes has been a focal issue in the fields of electrochemical sensors and biosensors [1,2,3]. Layer-by-layer (LbL) deposited polymer films have been widely used for modifying electrode surfaces because of the wide range of available polymer types: synthetic polymers [4,5,6], biopolymers such as proteins [7,8,9,10,11], oligosaccharides and polysaccharides [12,13], and polypeptides [14]. LbL film-modified devices have found application in sensors [15,16,17], stimuli-sensitive systems [18,19], and controlled release [20,21,22,23,24].

We have recently reported redox reactions of ferricyanide ions, Fe(CN)_6_^3−^, and hexaammine ruthenium ions, Ru(NH_3_)_6_^3+^, on gold (Au) electrodes modified with LbL films composed of anionic polysaccharides [25,26]. Redox reactions of Ru(NH_3_)_6_^3+^ and Fe(CN)_6_^3−^ significantly depend on the properties of the LbL films such as film thickness, surface charge, and type of polymer employed. It was also found that Fe(CN)_6_^3−^ is confined in LbL films composed of carboxymethylcellulose (CMC) and alginic acid (ALG) coupled with poly(amine)s. Confined Fe(CN)_6_^3−^ was successfully used as an electrocatalyst for the oxidation of ascorbic acid [27]. In contrast, redox reaction of Ru(NH_3_)_6_^3+^ in the CMC- and ALG-based LbL films is severely suppressed, suggesting that CMC- and ALG-based films contain net positive charge originating from ammonium groups in the poly(amine)s in the LbL films [25,26]. In this situation, it is interesting to study the voltammetric response of the redox ions on electrodes coated with LbL films where cationic polysaccharides have been used in place of anionic CMC and ALG. For this goal, in the present study, we have employed chitosan (CHI) as a prototype of cationic polysaccharides by coupling with poly(vinyl sulfate) (PVS) or poly(acrylic acid) (PAA) to construct LbL film-modified electrodes. CHI is a deacetylated product of the naturally occurring polysaccharide chitin; it has been attracting much attention as a component of LbL film because of its interesting properties such as biocompatibility, high swelling ability, and antibacterial activities [28,29,30,31]. Redox properties of Ru(NH_3_)_6_^3+^ and Fe(CN)_6_^3−^ on the CHI/PVS and CHI/PAA film-modified electrodes may be different from those on the CMC and ALG film-modified electrodes. In fact, we have found that Ru(NH_3_)_6_^3+^ is electrostatically confined in the LbL films whereas Fe(CN)_6_^3−^ is not. This result suggests that the CHI/PVS and CHI/PAA films contain net negative charge originating from the sulfonic acid and carboxylic acid groups in PVS and PAA, respectively. The present paper reports a significant effect from the net charge in the CHI-containing LbL films on the redox properties of Ru(NH_3_)_6_^3+^ and Fe(CN)_6_^3−^.

## 2. Experimental Section

### 2.1. Materials

**Figure 1:**
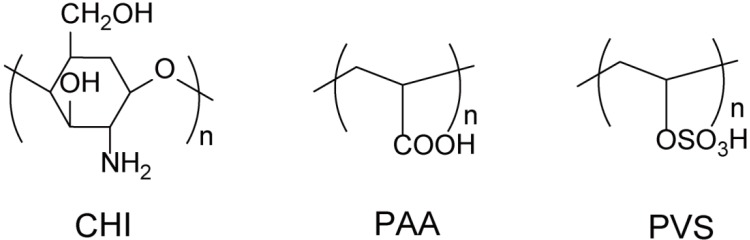
Chemical structures of chitosan (CHI); poly(acrylic acid) (PAA); and poly(vinyl sulfate) (PVS).

CHI (medium molecular weight) and PAA [average molecular weight (MW): 450,000] were purchased from Sigma-Aldrich Chemical Co. (St. Louis, MO, USA). PVS (MW: 240,000) and PEI (MW: 60,000–80,000) was purchased from Nacalai Tesque Co. (Kyoto, Japan). The chemical structures of polymeric materials used are illustrated in Figure 1. Sodium 3-mercapto-1-propanesulfonate (MPS) was purchased from Tokyo Kasei Co. (Tokyo, Japan). All other reagents were of the highest grade available and were used without further purification.

### 2.2. Apparatus

A quartz-crystal microbalance (QCM, QCA 917, Seiko EG & G, Tokyo, Japan) was used for gravimetric analysis of LbL films. A 9 MHz AT-cut quartz resonator coated with a thin Au layer (surface area: 0.2 cm^2^) was used as a probe, in which adsorption of 1 ng of substance induces a −0.91 Hz change in resonance frequency. Atomic force microscope (AFM, SPM-9600, Shimadzu Co., Kyoto, Japan) was used for imaging the surface of LbL films. All electrochemical measurements were carried out using an electrochemical analyzer (660B, ALS Co., Tokyo, Japan).

### 2.3. Preparation of LbL Film-Coated Electrodes

LbL films were prepared on the surface of an Au disk electrode (diameter: 3 mm), according to the reported procedure [25,26]. Preparation is briefly described here. The surface of the Au electrode was polished thoroughly using alumina slurry and rinsed in distilled water before use. The polished Au electrode was treated electrochemically in a 0.5 M H_2_SO_4_ solution by scanning the potential from −0.2 to 1.5 V *vs.* Ag/AgCl at scan rate of 0.1 V∙s^−1^ for 15 min. The cleaned Au electrode was first modified with MPS by immersing the electrode in a 5 mM MPS aqueous solution for 12 h. The surface of the MPS-modified Au electrode was modified with a PEI precursor layer by immersing the electrode in a 0.5 mg∙mL^−1^ PEI aqueous solution for 15 min and rinsing. The PEI-modified electrode was further modified with LbL film by dipping it alternately in a 0.5 mg∙mL^−1^ PVS or PAA solution (10 mM acetate buffer containing 150 mM NaCl, pH 3.0; or 10 mM 2-morpholinoethenesulfonic acid buffer containing 150 mM NaCl, pH 7.0) and a 0.5 mg∙mL^−1^ CHI solution (0.5% acetic acid containing 150 mM NaCl, pH 5.0) for 15 min with an intermediate 5 min rinse in the working buffer. The above procedure was repeated for depositing each desired layer of LbL film on the electrode.

### 2.4. Gravimetric Analysis of LbL Films

The surface of Au-coated quartz resonator was cleaned electrochemically in 0.5 M H_2_SO_4_ as described above. LbL films were deposited on both surfaces of the quartz resonator in a manner similar to the film deposition on the Au electrode. The film-coated probe was rinsed in pure water for 1 min and dried in air after each deposition until the resonance frequency showed a steady-state value to estimate the weight of the film.

### 2.5. AFM Imaging

AFM images of the surface of LbL films were recorded in air at room temperature using a SPM-9600 instrument operating in dynamic (tapping) mode. The sample for AFM observation was prepared on a glass slide.

### 2.6. Electrochemical Measurements

The electrochemical response of the electrodes was measured in a glass cell using the LbL film-modified electrode as the working electrode, a platinum wire as a counter electrode, and a Ag/AgCl electrode (3.3 M KCl) as a reference electrode. All measurements were performed under air at room temperature (approximately, 20 °C).

## 3. Results and Discussion

### 3.1. Preparation of Chitosan-Containing LbL Films

**Figure 2:**
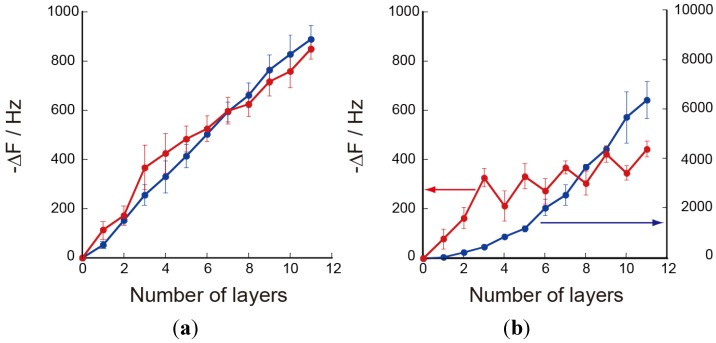
Frequency changes in the quartz-crystal microbalance (QCM) for deposition of (**a**) PEI(CHI/PVS)_5_ films; and (**b**) PEI(CHI/PAA)*_n_* films at pH 3.0 (blue) and pH 7.0 (red). Plotted points are averages of three measurements with standard deviation.

The deposition behavior of chitosan-containing LbL films was studied using a QCM. Figure 2a plots the decrease in the resonance frequency (−∆*F*) recorded upon depositing CHI/PVS films as a function of the number of depositions. The −∆*F* values increased in magnitude almost linearly as the number of depositions increased for the PEI(CHI/PVS)*_n_* films, suggesting that CHI and PVS were successfully deposited on the quartz resonator to form the LbL film. The effect of the pH of the PVS solution was rather small. It is clear that the PEI(CHI/PVS)*_n_* films formed through electrostatic affinity between positive charges on the CHI chains and negative charges on PVS. On the other hand, for the PEI(CHI/PAA)*_n_* films, the −∆*F* magnitude increased upon depositing CHI but was decreased upon depositing PAA, suggesting that some of the CHI located on the outermost surface of the film was desorbed during deposition of the next PAA layer (Figure 2b). Similar adsorption-desorption behavior has been reported for some LbL films [32,33]. Another feature of the PEI(CHI/PAA)*_n_* films is that the deposition behavior depended on the pH of the PAA solution. The −∆*F* values for the PEI(CHI/PAA)*_n_* film deposited from acidic PAA solution (pH 3.0) were markedly larger than those prepared using neutral PAA solution (pH 7.0), probably owing to protonation of carboxylate groups in PAA at pH 3.0. It is likely that protonated PAA assumes a coiled conformation in LbL films, resulting in thicker layers [5]. This is a clear contrast to the deposition behavior of PEI(CHI/PVS)*_n_* films, in which the −∆*F* values did not depend on the pH of the PVS solution. This is because sulfonic acid groups in PVS should be fully dissociated at both pH 3.0 and 7.0 owing to its strong acidity. The average thickness of the films prepared at pH 7.0 was estimated from the QCM data to be about 40 nm for the PEI(CHI/PVS)_5_ film and about 21 nm for the PEI(CHI/PAA)_5_ film in the dry state; these estimates assume that the frequency changes linearly depend on the deposited mass and the density of the films is near 1.2 g∙cm^−3^ [34].We used here QCM to qualitatively verify the deposition behavior of LbL films. It is known that the frequency changes in QCM depend not only on the deposited mass but also on other factors such as hydration and stiffness of the materials [35]. In fact, fluctuation in the −∆*F* values in our data is rather high. Nevertheless, the QCM results show that CHI-based LbL films could be successfully constructed through electrostatic binding between CHI and PVS or PAA.

**Figure 3:**
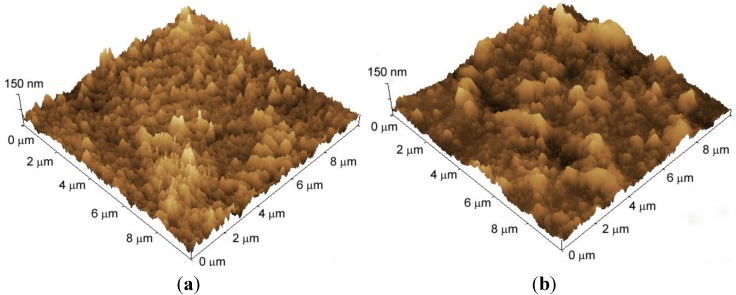
AFM three-dimensional images of (**a**) PEI(PAA/CHI)_5_; and (**b**) PEI(PVS/CHI)_5_ films. The films were prepared using pH 7.0 PAA and PVS solutions and pH 5.0 CHI solution.

The surface morphology of LbL films was studied using AFM. Figure 3 shows three-dimensional AFM images of the surface of dry LbL films. The surface is rather rough, with the root mean square (rms) roughness being 14.2 nm and 13.2 nm for the PEI(PAA/CHI)_5_ and PEI(PVS/CHI)_5_ films, respectively. Both the QCM and AFM results clearly show successful preparation of the CHI-containing LbL films.

### 3.2. Redox Reactions of Ru(NH_3_)_6_^3+^ and Fe(CN)_6_^3−^ on CHI/PVS and CHI/PAA Film-Modified Electrodes

**Figure 4:**
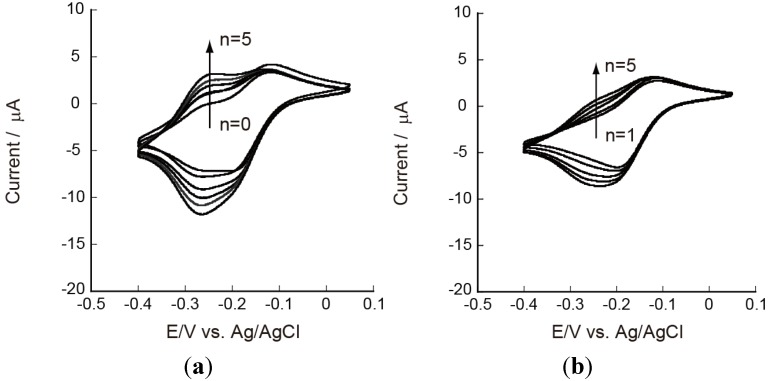
Cyclic voltammograms (CVs) of 5 mM Ru(NH_3_)_6_^3+^ on (**a**) PEI(PVS/CHI)*_n_*PVS; and (**b**) PEI(PVS/CHI)*_n_* films at pH 7.0.

**Figure 5:**
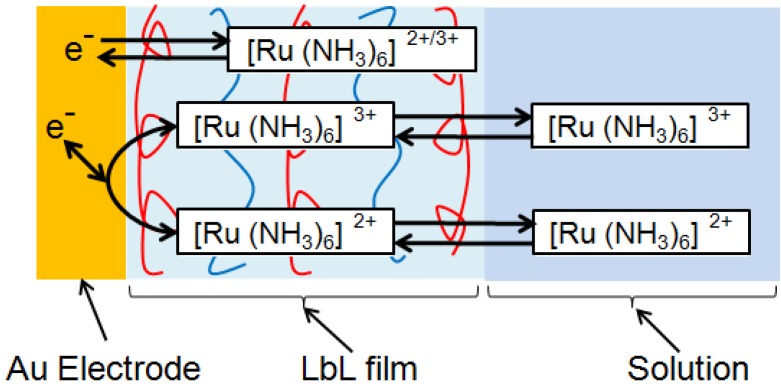
Schematic illustration of the redox reactions diffusing and confined Ru(NH_3_)_6_^2^^+/^^3^^+^ ions on the Au electrode.

Redox properties of CHI/PVS and CHI/PAA film-coated electrodes should be evaluated for their applications to electrochemical devices such as sensors and reactors. For this purpose, we have used Ru(NH_3_)_6_^3+^ and Fe(CN)_6_^3−^ as redox marker because these ions are known to be stable unless measurements are carried out in strongly acidic solutions [36]. In fact, these ions have been widely used as redox marker in voltammetric studies [37,38,39]. Figure 4 shows cyclic voltammograms (CVs) of Ru(NH_3_)_6_^3+^ on Au electrodes coated with PEI(PVS/CHI)*_n_*PVS and PEI(PVS/CHI)*_n_* films, where n denotes the number of bilayers in the LbL films. The CVs were recorded after the electrode had been immersed in a 1 mM Ru(NH_3_)_6_^3+^ solution for 15 min because the peak current in CVs increased with time during the first several minutes. For the PEI(PVS/CHI)*_n_*PVS film-coated electrodes (Figure 4a), redox peaks were observed in the potential range of −0.1 to −0.3 V; these peaks are attributed to redox reactions of Ru(NH_3_)_6_^3+^ [40,41]. The CV shows two oxidation peaks, at around −150 and −250 mV. The intensity of the latter peak increased as the number of bilayers was increased. The shape and intensity of the reduction peak was also dependent on film thickness: the peak at approximately −270 mV increased with an accompanying shoulder peak around −200 mV. These results suggest that two kinds of redox reactions take place concurrently in LbL films. It is worth noting that the separation between the anodic and cathodic peaks (∆*E*_p_) for the redox couple around −250 mV was small (10–20 mV), suggesting the CV peaks originate from Ru(NH_3_)_6_^3+^ confined in the films. In contrast, the ∆*E*_p_ values for the redox couple at −100 to −200 mV were 60–80 mV, showing that these peaks might have arisen from the redox reaction of diffusing species. Consequently, both the diffusing and confined species are involved in the redox reactions of Ru(NH_3_)_6_^3+^ in the PEI(PVS/CHI)_n_PVS films, as illustrated in Figure 5.

Similar CVs were observed on the electrodes coated with PEI(PVS/CHI)*_n_* films, whose outermost surfaces were covered with positively charged CHI (Figure 4b). These results show that the PEI(PVS/CHI)*_n_*PVS and PEI(PVS/CHI)*_n_* films are permeable to Ru(NH_3_)_6_^3+^ and that the ions are confined in the films, probably due to electrostatic attraction between positively charged Ru(NH_3_)_6_^3+^ and fixed negative charges in the films. Sulfonate residues in PVS should be responsible for the negative sites in the LbL films. It is likely that the amount of negative charge in PVS is higher than the amount of positive charge in CHI in the PEI(PVS/CHI)*_n_*PVS and PEI(PVS/CHI)*_n_* films, providing excess negatively charged sites for binding Ru(NH_3_)_6_^3+^. In this context, we have previously reported that LbL films composed of negatively charged polysaccharides (CMC and ALG) and synthetic poly(amine)s contain net positive charge originating from protonated amino groups in the poly(amine)s [25,26]. The results were rationalized in terms of higher charge densities in the poly(amine)s than in the polysaccharides. It is worth noting that despite a possible electrostatic repulsion between the ions and CHI, LbL films terminated with CHI are permeable to Ru(NH_3_)_6_^3+^, although the permeation was suppressed to some extent.

**Figure 6:**
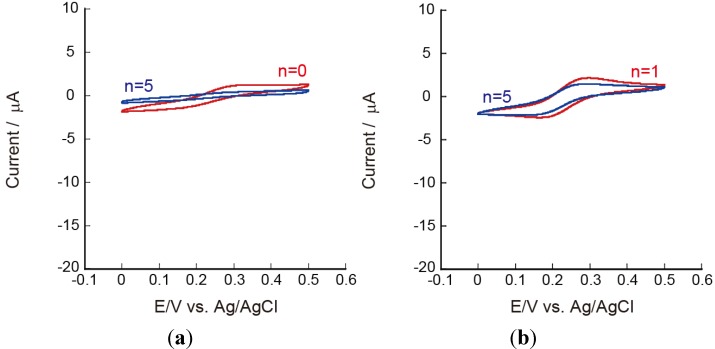
CVs of 5 mM Fe(CN)_6_^3−^ ions on (**a**) PEI(PVS/CHI)*_n_*PVS; and (**b**) PEI(PVS/CHI)*_n_* films at pH 7.0.

Figure 6 shows CVs of Fe(CN)_6_^3−^ on Au electrodes coated with PEI(PVS/CHI)_5_PVS and PEI(PVS/CHI)_5_ films. The redox reaction of Fe(CN)_6_^3−^ was strongly suppressed, suggesting that the LbL films are less permeable to negatively charged Fe(CN)_6_^3−^. These results support the view that the PEI(PVS/CHI)_5_PVS and PEI(PVS/CHI)_5_ films contain excess negative charges.

**Figure 7:**
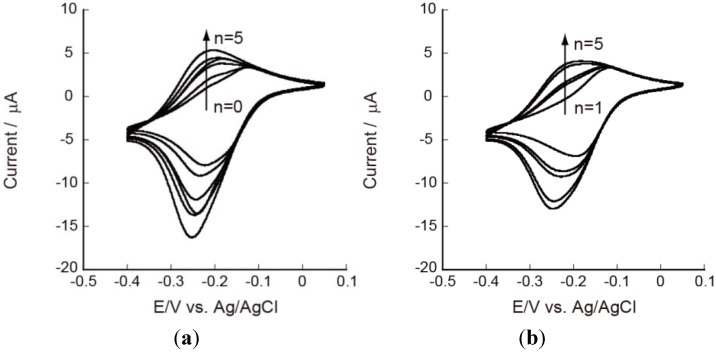
CVs of 5 mM Ru(NH_3_)_6_^3+^ on (**a**) PEI(PAA/CHI)*_n_*PAA; and (**b**) PEI(PAA/CHI)*_n_* films at pH 7.0.

The effects of type of polyanion in the film were studied using PAA in place of PVS. Figure 7 shows CVs of Ru(NH_3_)_6_^3+^ recorded on the PEI(PAA/CHI)*_n_*PAA and PEI(PAA/CHI)*_n_* film-coated electrodes. The characteristic features of the CVs were largely similar to those observed for the PVS-based films, except that in contrast to the PVS-based films, two anodic peaks were not clearly observed. Instead, the anodic peak current increased as the apparent *E*_p_ values shifted in the negative direction. The broad peaks might be composed of two kinds of redox peaks originating from diffusing and confined Ru(NH_3_)_6_^3+^.

### 3.3. Effects of pH on the Redox Reactions of Ru(NH_3_)_6_^3+^

**Figure 8:**
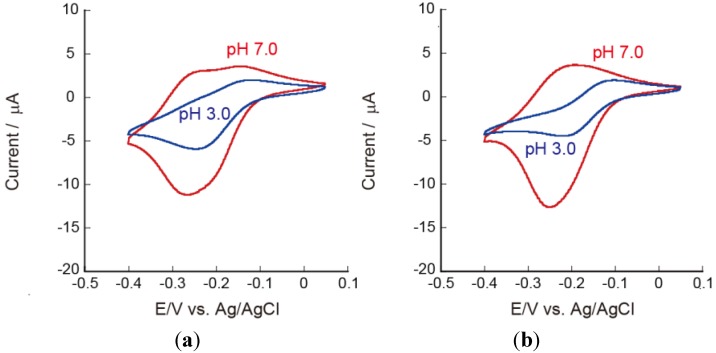
CVs of Ru(NH_3_)_6_^3+^ on (**a**) PEI(PVS/CHI)_5_; and (**b**) PEI(PAA/CHI)_5_ film-coated electrodes at pH 3.0 (blue) and pH 7.0 (red). The LbL films were prepared at pH 7.0.

It is interesting to evaluate the effects of pH on the redox reactions of Ru(NH_3_)_6_^3+^ on the LbL film-coated electrodes, because the amount of electric charge from CHI and PAA in the film should depend on the pH of the solution. In other words, the microenvironment in the LbL films is pH-dependent. Figure 8 shows the CVs of PEI(PVS/CHI)_5_ and PEI(PAA/CHI)_5_ film-coated electrodes in 0.5 mM [Ru(NH_3_)_6_]^3+^ solutions at pH 7.0 and 3.0. In this experiment, the PEI(PVS/CHI)_5_ and PEI(PAA/CHI)_5_ films were prepared using pH 7.0 PVS and PAA solutions. The anodic and cathodic currents in the CVs for the PEI(PVS/CHI)_5_ film-coated electrode were suppressed in the acidic solution (Figure 8a). The redox couple found at −250 to −300 mV at pH 7.0 was only weakly observed at pH 3.0. These results suggest that the amount of Ru(NH_3_)_6_^3+^ confined in the film is negligibly small at pH 3.0, probably due to increased number of positive charges as a result of protonation to amino groups in CHI. The CVs for the PEI(PAA/CHI)_5_ film-coated electrode also depended on the pH (Figure 8b). In the acidic solutions at pH 3.0, the CV exhibited typical diffusion-controlled waveforms, showing Ru(NH_3_)_6_^3+^ could not be confined at the acidic pH. This is attributed to the reduced number of negative charges in the PAA chains as well as the increased positive charge in CHI in the acidic solution. Thus, the PEI(PAA/CHI)_5_ film might not contain excess negative sites, meaning that no Ru(NH_3_)_6_^3+^ could be confined at pH 3.0.

**Figure 9:**
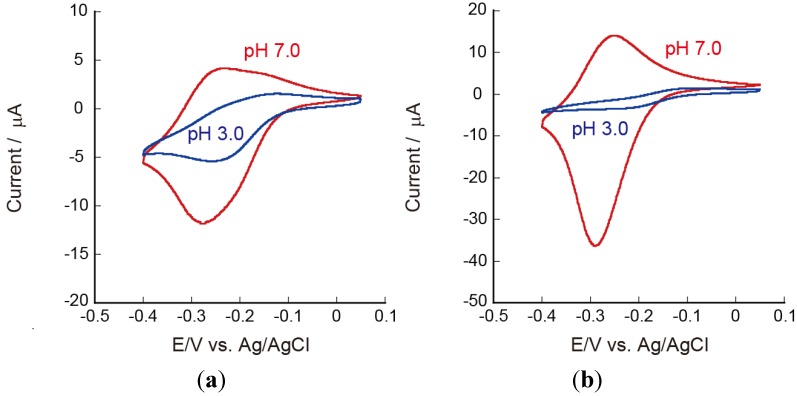
CVs of Ru(NH_3_)_6_^3+^ on (**a**) PEI(PVS/CHI)_5_; and (**b**) PEI(PAA/CHI)_5_ film-coated electrodes at pH 3.0 (blue) and pH 7.0 (red). The LbL films were prepared at pH 3.0.

The LbL films prepared using acidic PVS and PAA solutions may exhibit different properties from those prepared at pH 7.0. In particular, the pH-dependent QCM results (Figure 1) suggest that the effects may be significant for the PEI(PAA/CHI)_5_ films. Figure 9 shows the CVs of Ru(NH_3_)_6_^3+^ on the PEI(PVS/CHI)_5_ and PEI(PAA/CHI)_5_ film-coated electrodes, in which the LbL films were prepared using pH 3.0 PVS and PAA solutions. The CVs for the PEI(PVS/CHI)_5_ film-coated electrode were similar to those prepared using pH 7.0 solutions (see Figure 8a). It is likely that the PEI(PVS/CHI)_5_ films prepared at pH 3.0 and 7.0 bear a structural resemblance to each other; this would be consistent with the QCM results (see Figure 1). In contrast, for the PEI(PAA/CHI)_5_ film-coated electrode prepared at pH 3.0, the redox current was significantly higher than that recorded on the electrode prepared at pH 7.0. The higher redox response on the electrode prepared at pH 3.0 relates to thickness of the films. (The thickness of the PEI(PAA/CHI)_5_ film prepared at pH 3.0 is calculated by QCM to be about 300 nm, as compared to about 21 nm for the film prepared at pH 7.0). Consequently, a higher amount of Ru(NH_3_)_6_^3+^ can be immobilized in the thicker film, resulting in the enhanced redox response.

### 3.4. Stability of Ru(NH_3_)_6_^3+^ Confined in LbL Films

The stability of the Ru(NH_3_)_6_^3+^ confined in the LbL film was evaluated by monitoring the redox current in the CVs. The electrodes were stored in the working buffer at pH 7.0, containing either 10 or 150 mM NaCl when not being used for measurement. The peak current decreased rapidly immediately after preparation, suggesting a significant portion of confined Ru(NH_3_)_6_^3+^ was released rapidly out of the films. In the buffer solution with 150 mM NaCl, about 70% of the Ru(NH_3_)_6_^3+^ was released out of the film after 30 min. Thereafter, the release of Ru(NH_3_)_6_^3+^ from the film slowly continued, and only 5%–10% of the Ru(NH_3_)_6_^3+^ remained in the films after 20 h. The release of the ions was slightly suppressed in the medium containing 10 mM NaCl; 20%–30% of the Ru(NH_3_)_6_^3+^ was retained in the film after 20 h. This is probably because ion exchange between Ru(NH_3_)_6_^3+^ and Na^+^ in the film is suppressed in the 10 mM NaCl solution.

In a separate experiment, we found that uptake of Ru(NH_3_)_6_^3+^ in the LbL films was reversible. In fact, confinement of Ru(NH_3_)_6_^3+^ in the films could be done repeatedly by immersing the LbL film-coated electrodes in a 1 mM Ru(NH_3_)_6_^3+^ solution at pH 7.0 after Ru(NH_3_)_6_^3+^ had been fully released.

## 4. Conclusions

We have demonstrated that Ru(NH_3_)_6_^3+^ permeates LbL films composed of CHI and PVS or PAA, irrespective of the sign of electric charge on the film surface, suggesting that the CHI/PVS and CHI/PAA films contain net negative charge. Ru(NH_3_)_6_^3+^ could be confined in the LbL films, providing supporting evidence for the existence of excess negative charges in the LbL films. In contrast, Fe(CN)_6_^3−^ could not be confined in the LbL films due to a lack of electrostatic affinity to the films. Consequently, the present study, together with our previous studies [25,26], clearly demonstrates that the sign of net electric charge in polysaccharide LbL films can be tuned by an appropriate choice of polysaccharide type. Therefore, CHI-containing LbL films may be useful as scaffolds for immobilizing positively charged species, such as catalysts, on an electrode surface.
